# Greater body appreciation moderates the association between maladaptive attentional biases and body dissatisfaction in undergraduate women

**DOI:** 10.1177/2043808719838937

**Published:** 2019-04-16

**Authors:** Leah N. Tobin, Amy H. Barron, Christopher R. Sears, Kristin M. von Ranson

**Affiliations:** University of Calgary, Canada

**Keywords:** Body appreciation, body dissatisfaction, body image, attention, eye tracking

## Abstract

Attentional biases for weight-related information are thought to contribute to maintenance of body dissatisfaction and eating disorders. Women with greater body appreciation may pay less attention to thin-ideal cues if body appreciation protects them from negative effects of thin-ideal media, and if so, they may be less susceptible to development of maladaptive attentional biases. The present study used eye-gaze tracking to measure attention to weight-related words/images in 167 body-dissatisfied undergraduate women (aged 17–39 years) to examine the associations among body dissatisfaction, body appreciation, and attentional biases. Participants viewed displays of thin-related, fat-related, and neutral words/images while their eye fixations were tracked over 8-s intervals. We hypothesized body appreciation (as measured by the Body Appreciation Scale) would moderate the documented association between body dissatisfaction and attentional biases for thin-related information only, such that as body appreciation increased, the strength of the relationship between body dissatisfaction and attentional biases would decrease. Results indicated that body appreciation moderated the association between body dissatisfaction and attentional biases for thin-related words only. With low body appreciation, body dissatisfaction was positively associated with attention to thin-related words. With high body appreciation, there was an inverse association between body dissatisfaction and attention to thin-related words. Results suggest that body appreciation may be an effective prevention target for reducing maladaptive attentional biases.

Appearance is a core component of self-evaluation for girls and women (Stice & Bearman, 2001). Body dissatisfaction, considered one of the most robust and consistent eating pathology risk factors (Stice, 2002), is increasingly common in college women, with prevalence rates reported up to 80% (Neighbors & Sobal, 2007; Vohs, Heatherton, & Herrin, 2001). Body dissatisfaction is associated with several problematic health conditions and concerns. For example, body dissatisfaction is linked to depressive symptoms, low self-esteem, and emotional distress (Ohring, Graber, & Brooks-Gunn, 2002) and is a major risk factor for the development and maintenance of eating pathology (Fitzsimmons-Craft et al., 2012; Stice & Whitenton, 2002). Given the negative consequences associated with body dissatisfaction, it is important to investigate factors that can adversely affect body dissatisfaction as well as protective factors that can promote body satisfaction.

## Attentional biases related to body dissatisfaction

Cognitive theories of body dissatisfaction (Cash & Labarge, 1996; Williamson, White, York-Crowe, & Stewart, 2004) and eating disorders (Vitousek & Hollon, 1990) propose that appearance-, weight-, and shape-related schemas influence the processing of information related to body image. Maladaptive body image schemas are postulated to increase selective attention to body image-related information in the environment. Attentional biases related to body dissatisfaction have been well-documented, and eye-gaze tracking has been the dominant methodological paradigm used to assess attentional biases in body-dissatisfied individuals (Rodgers & DuBois, 2016). Attentional biases to body- and weight-related information in body-dissatisfied individuals have been identified in multiple studies (Gao et al., 2011; Markis & McLennan, 2011; Tobin, Sears, Zumbusch, & von Ranson, 2018; Withnell, Sears, & von Ranson, 2019). Researchers have found that body-dissatisfied women typically attend more to weight-related information, including both thin-related and fat-related information, compared to controls (Gao et al., 2014; Gao et al., 2013; Tobin et al., 2018; Withnell et al., 2019). For example, Tobin, Sears, Zumbusch, and von Ranson (2018) found that body-dissatisfied women gazed longer than body-satisfied women at both fat- and thin-related words over an 8-s presentation. Similarly, Gao et al. (2014) found that women with higher body dissatisfaction attended to images of thin and overweight bodies more than women with lower body dissatisfaction and were also faster to attend to these images. While body-dissatisfied women are found to gaze longer at both fat- and thin-related information, they may do so for different reasons. For example, a “fear of fatness,” which is hypothesized to play a large role in body dissatisfaction (Williamson et al., 2004), may be the cognitive factor underlying the heightened attention to fat-related stimuli (Rodgers & DuBois, 2016). Heightened attention to thin-related stimuli, on the other hand, may be more strongly related to the tendency of body-dissatisfied individuals to engage in upward social comparisons (Schaefer & Thompson, 2014).

Attentional biases may contribute to the development and maintenance of body dissatisfaction (Cash & Labarge, 1996; Williamson et al., 2004), a known risk factor for eating disorders (Stice, Marti, & Durant, 2011). Several studies have demonstrated a reciprocal relationship between body dissatisfaction and attentional bias, such that intentionally directing one’s attention to non-preferred appearance-related stimuli leads to increased body dissatisfaction (Joseph, 2014; Smeets, Jansen, & Roefs, 2011; Smith & Rieger, 2006). As there are numerous negative consequences associated with body dissatisfaction, it is important to study protective factors that may make individuals less susceptible to attentional biases related to body dissatisfaction. In the present study, we explored whether body appreciation is one such factor.

## Body appreciation

Thin-ideal media can lead to increased body dissatisfaction and eating disorder symptoms (Hawkins, Richards, Granley, & Stein, 2004). Body appreciation has been proposed to protect one from negative influences of thin-ideal media (Andrew, Tiggemann, & Clark, 2015). Unlike body satisfaction, body appreciation goes beyond the superficial physical attributes of the body; individuals with high levels of body appreciation do not base their self-worth on their outward appearance and are less likely to engage in social comparison (Homan & Tylka, 2018). Avalos, Tylka, and Wood-Barcalow (2005) identified four underlying contributors to body appreciation: holding positive evaluations/opinions of the body; body acceptance regardless of imperfections, shape, or weight; respecting and attending to bodily needs; and protecting the body by rejecting unrealistic appearance ideals. Body appreciation can predict additional variation in well-being after body satisfaction has been accounted for, and is therefore considered to be a distinct construct from body satisfaction (Avalos, Tylka, & Wood-Barcalow, 2005).

Several studies have concluded that higher levels of body appreciation can reduce negative effects of thin-ideal media exposure. For example, Andrew, Tiggemann, and Clark (2015) found that self-reported body dissatisfaction in women low in body appreciation increased following media exposure, whereas for women with higher levels of body appreciation this was not the case. Other studies have reported that compared to women with low levels of body appreciation, women with high levels of body appreciation engage in fewer dieting behaviors and place less importance on discrepancies between their own appearance and the appearance of models who reflect the thin-ideal (Andrew, Tiggemann, & Clark, 2016; Halliwell, 2013). Gillen and Dunaev (2017) found that women high in body appreciation placed more importance on expressing their own uniqueness than attempting to conform to media appearance ideals. Taken together, the literature points to growing evidence that greater body appreciation can protect women from the negative effects of media exposure.

Given that high levels of body appreciation are associated with smaller increases in state body dissatisfaction following exposure to thin-ideal media (Andrew et al., 2015), and that women with higher levels of body appreciation report fewer negative effects of media exposure (Halliwell, 2013), it is possible that women high in body appreciation pay less attention to thin-ideal cues, which then prevents these cues from influencing their state body dissatisfaction. Moreover, if body appreciation influences attentional biases related to body dissatisfaction, body appreciation may be an important protective factor to target in attention-related prevention efforts. For example, eating disorder preventive interventions have been found to increase self-reported body appreciation in high school girls (Halliwell, Jarman, McNamara, Risdon, & Jankowski, 2015), women over 25 with obesity (Olson, Thaxton, & Emery, 2018), and undergraduate men (Jankowski et al., 2017). Examination of the influence of body appreciation on maladaptive attentional biases related to body dissatisfaction provides direct evidence for the postulated moderation process whereby reduced attention to thin-ideal cues reduces the negative impact of thin-ideal media. This is the approach taken in the present study.

## The present study

The present study examined whether high levels of body appreciation moderate body-dissatisfied women’s attentional biases for thin-ideal cues. Body-dissatisfied women were the focus of the study because they are at heightened risk for negative influences of the media and have been shown to exhibit biases in attention to weight-related words (Gao et al., 2011; Tobin et al., 2018) and images (Cho & Lee, 2013; Gao et al., 2014; Withnell et al., 2019). Participant recruitment was therefore targeted to women with high levels of body dissatisfaction.

The design of our study allowed us to test several predictions. First, we predicted that our results would replicate previous findings that women with high levels of body dissatisfaction exhibit attentional biases for fat- and thin-related words and images. Specifically, we expected that body dissatisfaction would be positively associated with attentional biases for fat- and thin-related words and images. Second, we tested whether greater body appreciation made women less susceptible to attentional biases for thin-ideal–related cues associated with body dissatisfaction. Specifically, we predicted that body appreciation would moderate the relationship between body dissatisfaction and attentional biases for thin-related words and images: As body appreciation increased, the strength of the relationship between body dissatisfaction and attentional biases would decrease. We further predicted that at high levels of body appreciation, the association between body dissatisfaction and attentional biases for thin-related cues would be eliminated. We expected this moderation for thin-related cues only, given that high levels of body appreciation are associated with smaller increases in state body dissatisfaction following exposure to thin-ideal media (Andrew et al., 2015), and because women with high body appreciation are less likely to engage in social comparison (Homan & Tylka, 2018). Third, given that previous research has documented a protective role of body appreciation only for the harmful effects of thin-ideal media, we predicted that body appreciation would not moderate the relationship between body dissatisfaction and attention to fat-related words or images.

## Materials and methods

The study involved three phases. First, we collected ratings for a large set of images via an online survey to facilitate selection of stimuli to present to participants. Second, we screened prospective participants for study eligibility using an online prescreening survey. Third, eligible participants who signed up for the study visited the laboratory to complete an eye-tracking task and several questionnaires. These three phases are described in detail in the subsequent sections. The study was approved by the University of Calgary Conjoint Faculties Research Ethics Board.

### Stimuli development

#### Word stimuli

We used validated word stimuli from a previous study that examined attention to thin- and fat-related words in undergraduate women screened for body dissatisfaction (Tobin et al., 2018). In Tobin et al.’s study, female undergraduates enrolled in psychology courses completed an online survey. Each student was presented with 190 words to rate, which were randomly selected from a set of 380 words. Words were rated on category (the options were “thin,” “fat,” “neutral,” or “unsure”) and valence (from “very negative” to “very positive”). In total, 300 neutral, 40 thin-related, and 40 fat-related words were rated. To be eligible for use in Tobin et al.’s study, at least 70% of students had to agree to a word’s category (“thin,” “fat,” “neutral”) and less than 10% could be “unsure.” For the present study, the words meeting these requirements were used to create sets of four words that were presented to participants for an eye-tracking task. Each set of four words included one thin-related word (e.g., “lean”) or one fat-related word (e.g., “tubby”), and three neutral words (e.g., “humid”). The words in each set were matched on the number of letters and were similar in printed frequency (i.e., the number of times a word appears in print per million words). For example, one word set included the words: “massive” (seven letters; printed frequency 10.0), “intense” (seven letters; printed frequency 10.2), “lecture” (seven letters; printed frequency 10.5), and “twisted” (seven letters; printed frequency 10.6). Valence ratings were used to create sets of four words with similar valence. Refer to Tobin et al. (2018) for additional details.

#### Image stimuli

Adapting the methodology used by Gao et al. (2014), we used five categories of images: “fat” body images, “thin” body images, images of average-sized women, gardening-related images, and household-related images. All images were obtained from the Internet. To validate the images, an online survey was created and distributed using Qualtrics (www.qualtrics.com). One hundred undergraduate women provided informed consent and participated in exchange for bonus credit in a psychology course.

The survey presented participants with images from the five categories described earlier, one image at a time. The order in which the images were presented was randomly determined for each participant. The survey included 45 “thin-related” images that showed women with smaller bodies wearing either underwear or bathing suits, 45 “fat-related” images that showed women with larger bodies wearing either underwear or bathing suits, and 90 images of average-sized women engaged in various activities such as reading, talking, walking, or working on a computer; for these images, both body shape and weight information were less salient. Like Gao et al. (2014), we used images of average-sized women to distinguish between a specific attentional bias for fat- or thin-related information versus a general attentional bias for images of women. The control images were 70 images of gardening-related items and 70 images of household-related items. Each participant was presented with 106 or 107 images to rate, which were randomly selected (via Qualtrics) from a set of 320 images. Participants rated the valence of each image using a scale from −3 (“extremely negative”) to +3 (“extremely positive”), with a midpoint of 0. The “thin-related,” “fat-related,” and average-sized woman images were also rated on body size, using a scale from −3 (“extremely thin”) to +3 (“extremely fat”), with a midpoint of 0, and on attractiveness, using a scale from −3 (“extremely unattractive”) to +3 (“extremely attractive”), with a midpoint of 0.

Body size ratings were used to select the final set of stimuli. The 32 thin-related images with the lowest average rating of body size (i.e., those closest to “extremely thin”) were selected. The 32 fat-related images with the highest average rating of body size (i.e., those closest to “extremely fat”) were selected. The 64 images of average-sized women with body size ratings closest to 0 were selected. Sixty-four of the gardening-related images and 64 of the household-related images were selected from the original 70 images shown in the survey. The gardening-related and household-related images were selected to have similar valence ratings as the images of women, such that for each set of four images presented to participants, the four images had similar valence ratings.

### Eligibility screening

Prospective participants were recruited over a period of 10 months via an online prescreening survey. All participants provided written informed consent. Students were asked to rate their body dissatisfaction on an 11-point Likert-type scale from 0 (*extremely satisfied with your body*) to 10 (*extremely dissatisfied with your body*). This question was adapted from existing visual analog scale measures of appearance, weight, and body dissatisfaction (Heinberg & Thompson, 1995). Only women scoring in the top tertile of body dissatisfaction ratings were invited for a laboratory visit. A tertile split on body dissatisfaction scores has been used successfully to identify body-dissatisfied women in previous research examining attentional biases for body-related stimuli (Tobin et al., 2018). The top tertile of responses corresponded to ratings of 7–10 on the 11-point Likert-type scale.

### Participants

Inclusion criteria for participants were: (1) an undergraduate student at the University of Calgary enrolled in a psychology course, (2) self-reported identified female gender, (3) a score in the top tertile of body dissatisfaction scores on the online prescreening survey, and (4) self-reported to have no uncorrected visual impairment. Exclusion criteria for participants were: (1) participation in the online surveys used to develop the word and image stimuli used in the eye-tracking phase of the study and (2) under 17 years of age, determined via self-report. A total of 177 women signed up for a laboratory visit; 168 attended and participated in the study, and 1 participant was excluded from all analyses due to poor quality eye-tracking data, leaving a final sample of 167 women (mean body dissatisfaction score = 7.52, *SD* = .86, range = 7–10). The women ranged in age from 17 years to 39 years (mean age = 20.6). An a priori power calculation (G*Power 3.1; Faul, Erdfelder, Lang, & Buchner, 2007) determined a sample size of 77 was required to detect an anticipated medium effect size in the moderation analysis (i.e., linear multiple regression, fixed model, *R* ^2^ increase) with an α level of .05 and power at 80%. A medium effect size was chosen based on the effect sizes observed in previous research (Rodgers & DuBois, 2016; Tobin et al., 2018). Sociodemographic information and body mass index (BMI) of the sample are listed in Table 1.

**Table 1.** Sociodemographic characteristics and body mass index of the sample (*N* = 167).

Characteristic	*M* (*SD*, range)
Age (years)	20.62 (3.57, 17–39)
Body mass index (kg/m^2^)	23.82 (4.82, 16.13–44.63)
Year of undergraduate degree	%
First	34.1
Second	28.1
Third	19.2
Fourth	15.0
Fifth or more	3.6
Ethnicity	%
White	44.0
Southeast Asian	12.7
Chinese	10.8
South Asian	9.0
Arab/West Asian	6.6
Filipina	5.4
Latina	3.0
Black	3.0
Korean	1.8
Other	3.6

### Materials and measures

#### Demographic information

Demographic information including age, education, and ethnicity was collected via questionnaire.

#### Body mass index

Participants self-reported their heights and weights, which were used to calculate BMIs (kg/m^2^). BMI is an estimate of adiposity (Manson, Skerrett, & Willett, 2002). High correlations (*r* > .90) have been found between self-reported and measured weight and height (Gorber, Tremblay, Moher, & Gorber, 2007).

#### Body Shape Questionnaire

The Body Shape Questionnaire (BSQ; Cooper, Taylor, Cooper, & Fairbum, 1987) was administered to assess body dissatisfaction. This 34-item questionnaire assesses concern about body shape and the experience of “feeling fat” over the past 4 weeks. Each item uses a 6-point Likert-type scale ranging from “never” to “always.” Higher overall scores reflect greater levels of body dissatisfaction. The BSQ has good concurrent and discriminant validity (Cooper et al., 1987) as well as good reliability, with test–retest reliability of .88 (Rosen, Jones, Ramirez, & Waxman, 1996). Cronbach’s α for the current sample was .96.

#### Body Appreciation Scale 2

The Body Appreciation Scale 2 (BAS-2; Tylka & Wood-Barcalow, 2015) was administered to assess body appreciation. This questionnaire asks participants to rate 10 items on a 5-point Likert-type scale, ranging from “never” to “always.” Items probe feelings of respect for the body, being attentive to the needs of the body, appreciating the diverse and unique characteristics of the body, and feeling comfortable with the body. Higher overall scores reflect greater levels of body appreciation. The BAS-2 has good internal consistency, construct validity, and test–retest reliability in university women (Tylka & Wood-Barcalow, 2015). Cronbach’s α for the current sample was .93.

#### Eye-tracking paradigm

Eye gaze was tracked and recorded using an EyeLink 1000 eye-tracking system (SR Research Ltd., Ottawa, Ontario, Canada), which uses video-based infrared tracking technology. Eye movement data were collected at a 1,000-Hz sampling rate, which provided a temporal resolution of 2 ms. Average gaze error is less than 0.5° of visual angle. Stimuli were presented on a 24-in LCD monitor placed approximately 60 cm from the participant. To increase tracking accuracy, participants used a headrest while viewing the stimuli. A fixation marker was presented in the center of the display at the beginning of each trial, and stimuli were presented only when the eye-tracking system determined that the participant’s fixation was on the marker for at least 1 s, thereby ensuring that the participant was fixating on the center of the display before the stimuli were presented.

Each participant was shown 29 sets of four simultaneously presented words, and 32 sets of four simultaneously presented images. Each set was presented for 8 s. Eye-tracking data were collected throughout the 8-s presentations. The 29 sets of four words consisted of 12 thin-neutral word sets (one thin-related word and three neutral words), 12 fat-neutral word sets (one fat-related word and three neutral words), and 5 neutral word sets (four neutral words). The neutral word sets served to reduce the salience of the thin- and fat-related words in the other sets. As noted previously, these word sets were used in a previous study (Tobin et al., 2018). Words in the thin-neutral and fat-neutral sets were matched on number of letters and were selected to have similar printed frequencies and valence. The words in the neutral sets were matched to the words in the thin-neutral and fat-neutral sets on number of letters.

For the image sets, each participant was presented with 16 thin-neutral sets (one thin-related image, one image of an average-sized woman, one gardening-related image, and one household-related image) and 16 fat-neutral sets (one fat-related image, an image of an average-sized woman, one gardening-related image, and one household-related image). The images within each set had similar valence ratings, to ensure that valence did not influence participants’ attention to the different image types.

For each set of words and images, the four stimuli were placed in the four quadrants of the display (i.e., one word or image was presented in the top right, top left, bottom left, and bottom right of the display). Fat- and thin-related words/images were presented an equal number of times in each quadrant, to control for biases in the order in which the images were examined. There were two different blocks of word and image sets; the order of presentation of these blocks was counterbalanced across participants, and within each block, the presentation of the word and image sets was randomized. Together, there were 58 word sets and 64 image sets in total, but each participant saw a block that contained only half of these image and word sets. Data collection required approximately 10 min, including calibration of the eye-tracker.

### Procedure

Participants visited the laboratory in groups of three to five. At the start of the session, participants provided informed consent. Participants then completed several questionnaires via Qualtrics, including demographics questions, the BSQ, and the BAS-2. The questionnaires required approximately 15 min to complete. Eye-tracking data was collected in a private room dedicated for this purpose, and each participant was tested individually. The entire procedure was completed in 45 min to 1 hr. Participants were fully debriefed upon completion of the study.

### Data analysis

#### Data preparation

The EyeLink Data Viewer analysis software (SR Research) was used to process the fixation data, filtering for blinks, missing data, and other recording artifacts (using the default settings). To be included in the analyses, the minimum fixation duration was 100 ms; adjacent, sequential fixations that were less than 100 ms were merged into a single fixation. Each participant’s eye-tracking data had to meet quality checks to be included in the analyses. Attentional maintenance bias scores (%) were created for each participant by calculating the time spent fixating on an image or word type as a percentage of the total time spent fixating on the entire display over the 8-s presentations, averaged across all trials. For example, attentional maintenance bias scores for thin-related images were created by calculating the time spent fixating on thin-related images as a percentage of the total time spent fixating on the entire display over the 8-s presentations, averaged across trials. Higher scores therefore represent greater attentional maintenance (i.e., longer total fixation times).

#### Statistical analyses

Pearson’s bivariate correlations were used to determine whether body dissatisfaction was positively associated with attention to thin- and fat-related images and words (attentional maintenance bias scores), as reported previously (Gao et al., 2014; Gao et al., 2011; Tobin et al., 2018), and whether follow-up moderation analyses were appropriate. Bivariate correlations were also used to assess the associations between the study variables. Hierarchical multiple regressions were used to examine whether the relationship between body dissatisfaction and attentional maintenance bias scores for thin-related words and images was conditional on levels of body appreciation (demonstrating moderation). Significant associations between body dissatisfaction and attentional maintenance bias scores for the other stimuli (fat-related words and images, neutral words, images of an average-sized woman, gardening-related images, and household-related images) were also each followed up with hierarchical multiple regressions to determine whether associations were conditional on levels of body appreciation. Because no moderation was expected in this set of analyses, they served as control analyses.

Hierarchical multiple regression analyses were conducted using PROCESS (Hayes, 2012), a regression-based analytic tool available in SPSS Statistics, IBM, Version 24. A bootstrapping procedure (with 10,000 bootstrap resamples) and a product of coefficients approach were used to estimate bias-corrected and 95% confidence intervals, as described by Hayes (2013). This approach, carried out according to steps outlined by Hayes, balances statistical power and validity considerations (e.g., Type I and Type II errors). Body dissatisfaction scores (BSQ), body appreciation scores (BAS-2), and an interaction term between body appreciation and body dissatisfaction scores were included as predictors. Body dissatisfaction and body appreciation scores were mean-centered to avoid potentially problematic multicollinearity with the interaction term. The variance inflation factor for the two predictors was 1.56 (tolerance = .639), which indicated that multicollinearity was not a concern. Significant interaction effects were followed up with the Johnson–Neyman Regions of Significance (ROS) test to determine at what levels of the moderator variable (body appreciation) were there significant associations between body dissatisfaction and attentional maintenance bias scores.

## Results

### Analysis 1: Association between body dissatisfaction and attentional maintenance for thin- and fat-related stimuli

Table 2 lists the bivariate correlations between body dissatisfaction (BSQ scores) and attentional maintenance bias scores for thin-and fat-related words and images as well as for the neutral/control stimuli. As predicted, body dissatisfaction was positively associated with attentional maintenance bias scores for thin- and fat-related words and images—higher levels of body dissatisfaction were associated with greater attentional maintenance bias for these stimuli. Effect sizes were small to moderate (Cohen, 1992), which was expected, given the restriction in range for body dissatisfaction (i.e., the sample consisted entirely of women with high levels of body dissatisfaction). There was no association between body dissatisfaction and attentional maintenance bias scores for the images of average-sized women, and body dissatisfaction was negatively associated with the remaining neutral/control stimuli.

**Table 2.** Descriptive statistics for all study variables and Pearson’s correlations between self-report variables and attentional maintenance bias scores.

	Descriptive statistics		Pearson’s correlations	
Measure	*M*	*SD*	Body dissatisfaction	Body appreciation
Self-report				
Body dissatisfaction	108.31	31.84	—	−.601**
Body appreciation	3.08	0.77		—
Attentional maintenance bias scores				
Thin-related words	24.09	6.59	.169*	−.254**
Fat-related words	23.64	6.66	.316**	−.211**
Neutral words	23.24	1.87	−.211**	.232**
Thin-related images	31.93	12.69	.314**	−.343**
Fat-related images	31.32	11.14	.291**	−.220**
Images of average-sized women	25.79	5.40	−.004	.084
Gardening-related images	17.91	5.83	−.357**	.321**
Household-related images	19.12	6.44	−.244**	.188*

*p < .05; **p < .01.

Table 2 also lists the bivariate correlations between body appreciation (BAS-2 scores) and attentional maintenance bias scores for each type of word and image stimuli. Body appreciation was negatively associated with attentional maintenance bias scores for thin- and fat-related words and images. Body appreciation was positively associated with the remaining neutral/control stimuli, whereas for images of average-sized women, there was no association. As expected, body appreciation and body dissatisfaction were negatively correlated (–.601).

### Analysis 2: Moderation of attentional maintenance for thin-related words and images

Using hierarchical multiple regression analyses following the steps outlined earlier, body appreciation was examined as a moderator of the relationship between body dissatisfaction and attentional maintenance bias scores for thin-related words and thin-related images.

**Figure 1.**
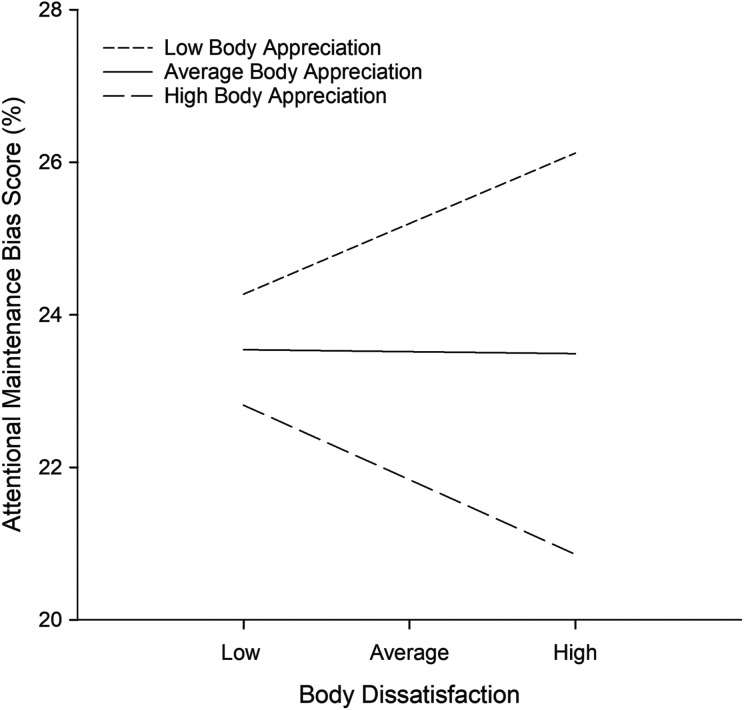
Associations between body dissatisfaction and attentional maintenance bias scores for thin-related words, at different levels of body appreciation. High and low values of body dissatisfaction and body appreciation represent scores 1 *SD* above and below the mean on the Body Shape Questionnaire and Body Appreciation Scale-2, respectively.

For attentional maintenance to thin-related words, the overall model including body dissatisfaction, body appreciation, and the interaction term between body dissatisfaction and body appreciation was significant, *R* ^2^ = .099, *F*(3, 163) = 5.99, *p* < .001. The addition of the interaction term to the model produced a significant increase in the variation accounted for, Δ*R* ^2^ = .03, *F*(1, 163) = 6.24, *p* = .014. Thus, as predicted, body appreciation was a significant moderator of the relationship between body dissatisfaction and attentional maintenance for thin-related words. The Johnson–Neyman ROS test indicated a significant positive association between body dissatisfaction and attentional maintenance bias scores for thin-related words at low levels of body appreciation (<1.67 on the BAS-2), no significant association at moderate levels of body appreciation, and a significant negative association at high levels of body appreciation (>4.8 on the BAS-2). These relationships are shown in Figure 1, which plots attentional maintenance bias scores for thin-related words at the mean, 1 *SD* above the mean, and 1 *SD* below the mean for levels of body dissatisfaction and body appreciation (calculated using PROCESS; Hayes, 2012).

For attentional maintenance to thin-related images, although the overall model including body dissatisfaction, body appreciation, and the interaction term between body dissatisfaction and body appreciation was significant, *R* ^2^ = .139, *F*(3, 163) = 8.79, *p* < .001, the addition of the interaction term to the model did not produce a significant *R* ^2^ increase, Δ*R* ^2^ = .00, *F*(1, 163) = 0.59, *p* = .444. Thus, contrary to our prediction, for thin-related images, body appreciation did not moderate the relationship between body dissatisfaction and attentional maintenance bias scores.

### Analysis 3: Moderation of attentional maintenance for fat-related words and images

Body appreciation was examined as a moderator of the relationship between body dissatisfaction and attentional maintenance bias scores for fat-related words and fat-related images using the statistical procedures described earlier.

For attentional maintenance to fat-related words, although the overall model including body dissatisfaction, body appreciation, and the interaction term between body dissatisfaction and body appreciation was significant, *R* ^2^ = .101, *F*(3, 163) = 6.10, *p* < .001, the addition of the interaction term to the model did not produce a significant *R* ^2^ increase, Δ*R* ^2^ = .00, *F*(1, 163) = 0.11, *p* = .740. Thus, as predicted, for fat-related words, body appreciation did not moderate the relationship between body dissatisfaction and attentional maintenance bias scores.

Similar results were obtained for attentional maintenance to fat-related images. The overall model including body dissatisfaction, body appreciation, and the interaction term was significant, *R* ^2^ = .094, *F*(3, 163) = 5.61, *p* = .001, but the addition of the interaction term to the model did not produce a significant *R* ^2^ increase, Δ*R* ^2^ = .01, *F*(1, 163) = 0.99, *p* = .321. Thus, for fat-related images, body appreciation did not moderate the relationship between body dissatisfaction and attentional maintenance bias scores.

### Analysis 4: Moderation of attentional maintenance for images of average-sized women, gardening-related images, and household-related images

In the final set of analyses, body appreciation was examined as a moderator of the relationship between body dissatisfaction and attentional maintenance bias scores for neutral words, images of average-sized women, gardening-related images, and household-related images, using the same statistical procedures described earlier. For all of these analyses no moderation effect was predicted.

For attentional maintenance to neutral words, the overall model including body dissatisfaction, body appreciation, and the interaction term between body dissatisfaction and body appreciation was significant, *R* ^2^ = .065, *F*(3, 163) = 3.78, *p* = .012, but the addition of the interaction term to the model did not produce a significant *R* ^2^ increase, Δ*R* ^2^ = .00, *F*(1, 163) = .53, *p* = .467. Thus, for neutral words, body appreciation did not moderate the relationship between body dissatisfaction and attentional maintenance bias scores.

For attentional maintenance to images of average-sized women, the overall model including body dissatisfaction, body appreciation, and the interaction term was not significant, *R* ^2^ = .023, *F*(3, 163) = 1.26, *p* = .288. As a result, tests for a moderation effect were not necessary.

For attentional maintenance to gardening-related images, the overall model including body dissatisfaction, body appreciation, and the interaction term was significant, *R* ^2^ = .147, *F*(3, 163) = 9.34, *p* < .001, but the addition of the interaction term to the model did not produce a significant *R* ^2^ increase, Δ*R* ^2^ = .00, *F*(1, 163) = 0.28, *p* = .598. Thus, for gardening-related images, body appreciation did not moderate the relationship between body dissatisfaction and attentional maintenance bias scores.

Finally, for attentional maintenance to household-related images, the overall model including body dissatisfaction, body appreciation, and the interaction term was significant, *R* ^2^ = .068, *F*(3, 163) = 3.96, *p* = .009, but the addition of the interaction term to the model did not produce a significant *R* ^2^ increase, Δ*R* ^2^ = .01, *F*(1, 163) = 1.00, *p* = .320. Thus, for household-related images, body appreciation did not moderate the relationship between body dissatisfaction and attentional maintenance bias scores.

## Discussion

This study examined the relationships among body dissatisfaction, body appreciation, and attentional biases for thin- and fat-related words and images in a large sample of body-dissatisfied undergraduate women. The purpose of our study was to determine whether higher levels of body appreciation moderated the association between body dissatisfaction and attentional biases for thin-ideal cues. Understanding the influence of body appreciation on attentional biases has implications for prevention programs seeking to protect women from the negative influences of thin-ideal media (Andrew et al., 2015).

### Associations among body dissatisfaction, body appreciation, and attentional maintenance

For thin-related images and fat-related words, the analyses revealed a moderate, positive relationship between body dissatisfaction and attention to these stimuli, and a weak, positive relationship between body dissatisfaction and attention to fat-related images and thin-related words. Consistent with previous research (Gao et al., 2014; Tobin et al., 2018; Withnell et al., 2019), these findings indicate that greater body dissatisfaction is associated with heightened attention to weight-related information. These associations are especially notable given the restriction of range in body dissatisfaction in the sample, which consisted only of women reporting high body dissatisfaction. These results contribute to a growing body of evidence that body dissatisfaction is associated with maladaptive attentional biases that contribute to the persistence of negative body image (Rodgers & DuBois, 2016). Because exposure to thin-ideal cues contributes to body dissatisfaction (Shroff & Thompson, 2006), the reciprocal role of these etiological and maintenance factors highlights the importance of body dissatisfaction prevention efforts.

The present study extended previous research by examining the association between body appreciation and attention to weight-related information. Analyses revealed a moderate inverse relationship between body appreciation and attention to thin-related images, and a weak inverse relationship between body appreciation and attention to thin-related words, fat-related images, and fat-related words. These findings indicate that higher levels of body appreciation are associated with reduced attention to weight-related information. Our results are consistent with questionnaire-based research that suggests that high levels of body appreciation protect women from the negative influences of thin-ideal media (Andrew et al., 2015).

We found that body dissatisfaction and body appreciation were moderately correlated (*r* = –.601). The strength of the association is similar to that found between body appreciation and measures of body satisfaction in previous research (Avalos et al., 2005). It is important to note that while these constructs are related, Avalos et al. found that body appreciation predicted additional variation in measures of well-being (e.g., self-esteem, optimism) above and beyond other measures of body image, including body dissatisfaction, when entered incrementally in a hierarchical regression. The correlation we observed in our sample lends support to the conclusion that body appreciation and body satisfaction are related but distinct constructs.

### Moderating role of body appreciation on the relationship between body dissatisfaction and attention to thin-related words

As predicted, body appreciation moderated the association between body dissatisfaction and attention to thin-related words. For women with low levels of body appreciation, the association between body dissatisfaction and attention to thin-related words was positive, with higher levels of body dissatisfaction associated with greater attention to these words. For women with average levels of body appreciation, body dissatisfaction was unrelated to attention to thin-related words, suggesting that average body appreciation buffered the association between body dissatisfaction and attention. Given that research has shown that body appreciation protects women against the negative influences of thin-ideal media (Andrew et al., 2015), these findings provide preliminary support for the interpretation that body appreciation may partially exert its effect by influencing women to pay less attention to thin-ideal cues, making them less likely to be negatively affected by these cues. The absence of a moderation effect for fat-related words and images, and for all neutral stimuli (neutral words, images of average-sized women, gardening-related images, and household-related images), highlights the uniqueness of the moderation effect for thin-related words.

The moderation effect revealed that for women with very high levels of body appreciation (at or above a score of 4.8 on the BAS-2 scale), the association between body dissatisfaction and attention to thin-related words was negative (i.e., attention to these words decreased as body dissatisfaction increased). One explanation for this outcome is that women with high levels of body appreciation and body dissatisfaction avoid thin-related information. For example, thin-ideal information may become more feared in body-dissatisfied women (Rodgers & DuBois, 2016), or more threatening, triggering negative weight-related self-schemas (Vitousek & Hollon, 1990). Women with high levels of body appreciation may avoid this information because it is threatening, given that a key characteristic of high body appreciation is the protection of body image by rejection of unrealistic appearance ideals (Avalos et al., 2005). If the combination of high body dissatisfaction and high body appreciation leads to such avoidance, then this has implications for the interpretation of mixed results in the attentional bias literature (Rodgers & DuBois, 2016). That is, some researchers have found that women high in body dissatisfaction avoid thin-related information (Gao et al., 2011; Glauert, Rhodes, Fink, & Grammer, 2010), whereas others have found that women high in body dissatisfaction exhibit heightened attention to thin-related information (Gao et al., 2014; Tobin et al., 2018). It is possible that body appreciation acts as a confounding third variable in studies of body-dissatisfied women by influencing their habitual patterns of attention to thin-ideal stimuli, leading to an avoidance of thin-related stimuli in some body-dissatisfied individuals and preferential processing of the same stimuli in others.

Although body appreciation may protect body-dissatisfied women from preferential processing of lexical information related to the thin-ideal by buffering the association between body dissatisfaction and attention for thin-related words, at very high levels of body appreciation it is unclear whether the observed avoidance of thin-related information reduces body dissatisfaction or future onset of eating pathology. It is possible that avoidance of threatening stimuli could contribute to maintenance of body dissatisfaction if the avoidance interferes with the efficacy of a treatment approach (e.g., prevention efforts aimed at directly improving body dissatisfaction; Stice, Rohde, & Shaw, 2013). It is essential for future research to examine the outcomes of attentional avoidance versus attentional maintenance of thin-related words on body dissatisfaction and eating disorder risk factors longitudinally, to determine appropriate attentional targets for prevention.

### Body appreciation and the relationship between body dissatisfaction and attention to thin-related images

Contrary to our prediction, body appreciation did not moderate the association between body dissatisfaction and attention to thin-related images. Important differences between the way in which word and image stimuli are processed may partially account for this discrepancy. Word stimuli may resemble one’s inner discourse and be interpreted as self-relevant information (Rodgers & DuBois, 2016). According to cognitive models, negative weight-related schemas are maintained through selective processing of self-relevant information that perpetuate the schema (Vitousek & Hollon, 1990). Thus, for those with heightened body dissatisfaction, thin-related words may be more schema relevant and therefore more likely to be preferentially processed, with body appreciation moderating this association.

For image stimuli, however, these relationships are likely more complicated. For body-dissatisfied women, attention to thin-ideal images may be more strongly affected by the influence of social comparison than attention to thin-ideal words. Social comparison has been proposed as one of the principal mechanisms through which thin-ideal media increases body dissatisfaction (Thompson, Coovert, & Stormer, 1999). Upward social comparisons (i.e., to more attractive targets) may be more strongly involved when body-dissatisfied women view images of thin women, because images are richer stimuli that have a closer resemblance to the thin-ideal messages women regularly encounter in advertisements and social media. Another difference between thin-related images and thin-related words is that many thin-related words have more than one meaning (e.g., “lean”); for these words, an upward social comparison may not be triggered immediately, in which case it may have little or no affect on attention. If greater body appreciation does not protect women from upward social comparisons when viewing body images, it would explain why body appreciation did not influence the association between body dissatisfaction and attention to thin-related images. The potential influence of social comparison in our study points to an important direction for future research; namely, an examination of the role that social comparison plays in the manifestation of attentional biases related to body dissatisfaction. The investigation of social comparison processes in this context has been limited (Rodgers & DuBois, 2016).

### Strengths and limitations of the present study

Strengths of the study included a large sample size, an a priori power calculation to ensure sufficient power to detect a moderation effect, use of eye-tracking technology to assess attention, the use of image and word stimuli validated in a sample of university women, the opportunity to confirm previous findings of attentional biases in body-dissatisfied women, and the assessment of a moderating variable in the association between body dissatisfaction and attentional biases for thin-ideal cues.

Our findings should be interpreted in the light of limitations of the study. We used a convenience sample of female university students, limiting the generalizability of the results to younger women fluent in English who are attending university. BMI was assessed via selfreported weight and height, and while highly correlated (Gorber et al., 2007), research has shown that self-report can result in underestimation of weight and overestimation of height (Krul, Daanen, & Choi, 2010), which reduces calculated BMI. We used cross-sectional assessments of body dissatisfaction, body appreciation, and attention, whereas longitudinal research is necessary to determine whether body appreciation can prevent maintenance or worsening of maladaptive attentional biases. On the other hand, this study has outlined a methodology for future studies that could use a longitudinal design to explore the effects of body appreciation on attention to weight-related stimuli. An additional limitation is that we did not assess for a current or past eating disorder, and so we are not able to determine what impact an eating disorder may have had on our results. By using a nonclinical sample, we may have underestimated the relations among body dissatisfaction, body appreciation, and attentional bias. Research has documented stronger attentional biases for weight-related stimuli in clinical populations of women diagnosed with an eating disorder (Dobson & Dozois, 2004). On the other hand, because the entire sample consisted of women with high levels of body dissatisfaction, a known risk factor for eating disorders (Stice et al., 2011), the pattern of attentional biases observed may be representative of clinical samples. Future research should explore whether body appreciation reduces attentional biases in individuals with an eating disorder.

## Conclusions

The present study is the first to demonstrate a moderating role of body appreciation on the association between body dissatisfaction and attention to thin-related words. It is also the first to demonstrate a relationship between body appreciation and attention to thin- and fat-related images and words, suggesting that as body appreciation increases, attention to these weight-related stimuli decreases. Our results show that body appreciation is a relevant factor in biased information processing related to body image. With respect to clinical implications, our results suggest that body appreciation may be an effective target for prevention efforts aimed at reducing maladaptive attentional biases associated with body dissatisfaction and eating disorders. Longitudinal research will be necessary to determine whether high levels of body appreciation protect women from developing attentional biases for weight-related information and whether the influence of body appreciation on attentional biases inhibits the onset of other eating disorder risk factors.
